# Chromosomal scale assembly and functional annotation of the apicomplexan parasite *Eimeria acervulina*

**DOI:** 10.1038/s41597-025-04653-1

**Published:** 2025-05-23

**Authors:** Subodh K. Srivastava, Carolyn C. Parker, Peter C. Thompson, Matthew S. Tucker, Benjamin M. Rosenthal, Asis Khan, Matthew J. Valente, Mark C. Jenkins

**Affiliations:** 1 USDA ARS, Animal Parasitic Diseases Laboratory, Beltsville Agricultural Research Center, East, Building 1040, 10300 Baltimore Ave., Beltsville, MD 20705 USA; 2 College of Osteopathic Medicine, Lake Erie College of Osteopathic Medicine, 5000 Lakewood Ranch Blvd, Bradenton, FL 34202 USA

**Keywords:** Zoology, Genome assembly algorithms

## Abstract

Apicomplexan parasites are single-celled obligate intracellular eukaryotic organisms that cause significant animal and human disease and pose a substantial health and socioeconomic burden worldwide. *Eimeria acervulina* is one such parasite of chickens, representative of several *Eimeria* species causing coccidiosis disease. A complete assembly of the *E. acervulina* genome may help identify markers of drug-resistance and design recombinant vaccines. We sequenced *E. acervulina* APU1 strain using Oxford Nanopore Sequencing and Illumina technology in combination with a Hi-C (Omni-C) proximity linkage library and achieved a chromosomal scale assembly using the MaSuRCA assembler. The final assembly was 52 Mb. with 15 chromosomes and 99% BUSCO completeness. A total of 7,621 genes were predicted using a pipeline of BRAKER3, GeneMark-ETP and AUGUSTUS, of which 4,647 (60.97%) have a predicted Pfam function and 1,962 (25.74%) have Gene Ontology (GO) terms matching molecular, biological, and functional classes. Stage-specific transcriptome analysis revealed 9,761 transcripts. This genome assembly and transcriptome analysis provides the foundation for identifying biologically important candidates for anticoccidial drug and vaccine development.

## Background & Summary

Apicomplexan parasites in the genus *Eimeria* cause enteric disease in livestock. Among livestock hosts, 12 *Eimeria* spp. have been described from cattle, 11 species from sheep, 9 from goats, 7 from turkeys and 7 from chickens^1,2^. Avian coccidiosis, widespread in poultry, causes more than $14 billion in economic damage each year to the poultry industry, worldwide^3,4^. This parasite invades epithelial tissues of the intestine, causing severe damage in birds and predisposes them to a potentially fatal infection called necrotic enteritis, leading to significant economic losses^5^.

*Eimeria acervulina* shares close similarity with other apicomplexan species that impair human and animal health^6^. In addition to its importance in chicken husbandry, it has utility as a surrogate organism for human parasites in the genus *Cyclospora* for basic and applied research applications^7–10^, addressing longstanding research gaps in less easily studied species^7^. Consequently, we sought a chromosomal scale assembly of *Eimeria acervulina* to establish its genetic architecture and provide insights relevant to parasite control.

**Fig. 1:**
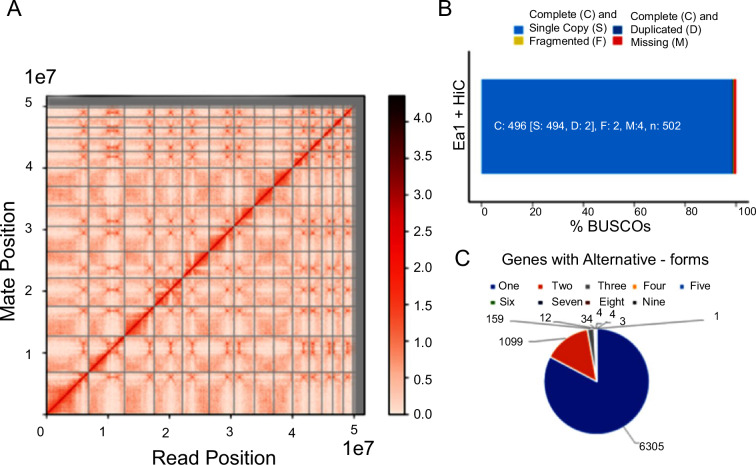
(**A**) Hi-C interaction matrix of *de novo* assembly contigs and Dovetail OmniC read pairs. Scaffolding was based on a likelihood model for genomic distance between read pairs which was used to identify and break putative misjoins, to score prospective joins, and make joins scaffold into chromosomes. (**B**) BUSCO analysis showed 99% completeness with 496 gene-sets out of 502 groups identified. Of these, 494 genes were complete and single copy, 2 complete and duplicated and, 2 fragmented and 4 missing genes. (**C**) Predicted genes with RNA-Seq shows 7,621 genes in *E. acervulina* APU1 with 1,316 genes showing alternative forms. (**D**) The Cluster of Orthologous Gene (COG) analysis found that 30.12% (1,899/6,305) single-copy genes have functional categories.

To achieve a high-quality assembly, we used multiple sequencing technologies and a hybrid assembly approach. We used three different technologies to assemble this genome at chromosomal scale using Oxford Nanopore long reads (ONT using MinION™) (1,183,636 reads with a mean read length of 5k), Illumina NextSeq. 2000 short reads (78 million reads) and 18 million reads of Hi-C data (Omni-C) (Table 1). The hybrid mode MaSuRCA^11^ assembler produced contiguous sequences of 51.70 Mb containing 7,621 genes. The chromosomal scale assembly (Fig. 1A) was achieved by scaffolding with approximately 30X Hi-C sequence coverage produced on an Illumina sequencer and analysed with HiRise^12^.

**Table 1:** Summary and type of obtained sequencing data generated and used in assembly and annotation of *E. acervulina* APU1.

Sequencing libraries	Total data	Mean Read Length (bp)	Coverage (X)
Nanopore (ONT)	1,183,636	5249	120
HiSeq-2000 PE	78 M	150	250
Hi-C (OminiC) PE	17.8 M	150	30
RNA-Seq	50 M	75	—

The genome completeness and quality were evaluated with Benchmarking Universal Single-Copy Orthologs (BUSCO), revealing 98.8% completeness (496) based on the 502 coccidia orthoDB v10 ortholog set. Of these, 494 genes were complete and single copy, 2 complete and duplicated, 2 fragmented, and 4 missing^13,14^ (Fig. 1B). Further analysis of gene prediction and annotation uncovered 7,621 genes with 6,305 primary genes and 1,316 designated as alternative forms^15^ (Fig. 1C). Annotation based on the Pfam v35.0 database with 19,633 entries and found 4,647 genes with Pfam functions and gene ontology (GO) terms with 1,962 classified into molecular, biological, and functional groups^16,17^. The best assemblies can include regions that have evolved through adaptations in paralogous sequences created by duplication. We compared our assembly to the previously published *Eimeria acervulina* Houghton assembly, which reported a 45.6 Mb genome comprising 3,415 scaffolds and 6,867 genes^18^. Our assembly increases the number of predicted genes by 11% and the total chromosomal assembly length by 12%, adding 6 Mb. The gene-to-gene comparison revealed 1,729 unique to the new APU1 assembly; 5,892 (77%) matched candidates in each isolate; their functional predictions are presented in Table S1.

This chromosomal scale assembly forms the basis for additional studies and facilitates an understanding of chromosomal rearrangement and genome structure. Furthermore, fine variations of gene and non-genic sequences may be examined for molecular, biological and functional differences that are involved in metabolism, growth and development of these organisms. Comprehending the genome biology of *E. acervulina* supports development of new drugs and vaccines to improve global food security^18^. We anticipate that this blueprint of chromosomal scale genome and functional analysis will hasten development of cost-effective vaccine candidates and facilitate biological research aimed at understanding parasite prevalence and preventing and controlling *E. acervulina* infections.

## Methods

### Sample collection and sequencing

*E. acervulina* oocysts were collected from litter on a commercial broiler farm in Maryland, USA and cloned by limiting dilution. These oocysts have since been propagated, as the APU1 strain, every 3-4 months in chickens at APDL, USDA, ARS, Beltsville for over 35 years. For DNA extraction, Oocysts are sporulated using standard procedures followed by storage in 2% K_2_CrO_4_ at 4°C. DNA extraction and preparation of sequencing libraries were carried out using methods developed in our laboratory^3^. *Eimeria acervulina* sporozoites were excysted from sporocysts that were released by grinding oocysts^19,20^. The sporozoites were centrifuged at 4000 rpm (2100 g) for 10 minutes, washed with saline A (0.14 M NaCl, 5 mM KCl, 4.2 mM NaHCO_3_, 0.1% glucose, pH 7.0), resuspended in 500 μl InhibitEX Buffer prior to DNA extraction using the QIAmp Fast DNA Stool Mini Kit according to manufacturer’s instructions (Qiagen, Germantown, MD). The integrity of DNA was analysed with Genomic DNA Screen Tape on a Tape Station instrument (Agilent Technologies, Santa Clara, CA) showing a DNA Integrity Number of 6.3 with peak size of 11.1 kb.

We first sequenced the genome of this isolate via long-read sequencing (Oxford Nanopore Technologies (ONT), Oxford UK) using 1 µg of genomic DNA and the ONT ligation sequencing kit SQK-LSK110. Approximately 200 ng of total library was sequenced for 48 h on a MinION flow cell (R9.4.1) per manufacturer recommendations^3,21^. To collect an adequate number of sequences, we repeated the sequencing twice and collected approximately 7 Gb of long read sequence data. We used Nanoplot and NanoStat software to evaluate the quality of sequencing results^22,23^. The sequence basecalling, translating raw electrical signal of Nanopore Sequencing to nucleotide sequence, influence the quality of sequences produced by Oxford Nanopore Technologies (ONT)^24^. We used the Guppy basecaller with the high accuracy model for basecalling purposes^24^. A complementary short-read dataset was produced on an Illumina NextSeq, employing an Illumina DNA Prep kit (Illumina, USA) with dual-indexed paired end Illumina indexes for sample identification^3^. Thirdly, contiguity was improved by interrogating chromatin interactions among physically proximate portions of the genome using Hi-C technology (Omni-C, CantataBio, Scotts Valley, CA)^3,25,26^.

To construct a library for proximity mapping using Omni-C, we excysted sporozoites *in vitro* and purified them using 5 µm PluriStrainer filters (Pluriselect, El Cajon, CA). The Hi-C library was prepared using the manufacturer’s instructions of their Dovetail Omni-C kit, version 2.0 (CantataBio, Scotts Valley, CA). Chromatin was fixed in place with formaldehyde in the nucleus and then extracted and digested with DNase I (at a further 1:10 dilution of the original Dovetail protocol for only 2 min instead of 30 min). Chromatin ends were repaired and ligated to a biotinylated bridge adapter followed by proximity ligation of adapter containing ends. After proximity ligation, crosslinks were reversed, and the DNA purified. The purified DNA was treated to remove biotin that was not internal to ligated fragments. Sequencing libraries were generated using NEB Next Ultra enzymes (New England Biolabs, Ipswich, MA) and Illumina-compatible adapters. Biotin-containing fragments were isolated using streptavidin beads before PCR enrichment of each library. The library was then sequenced on an Illumina HiSeqX platform to produce approximately 30X sequence coverage. Reads with mapping quality (MQ) greater than 50 were used for scaffolding (Fig. 1A).

### Genome assembly and scaffolding with HiRise

We initially produced draft genome assemblies with the long reads derived from Oxford Nanopore Sequencing and the short reads derived from Illumina sequencing using the hybrid assembly capabilities of MaSuRCa. The draft assembly had 114 contigs with an N50 of 1,141,052 base pair (bp). Dovetail OmniC library sequences were aligned to the draft input assembly using bwa^27^. The separations of Dovetail OmniC with 13,518,859 read pairs mapped within draft scaffolds were analysed by HiRise. HiRise produced a likelihood model for genomic distance between read pairs, identified putative mis-joins, scored prospective joins, and ultimately made joins among contigs that scored above a threshold (weakspots -q 50 and export link -C16 resulted 50 scaffolds). The final chromosomal scale scaffolds were oriented using available chromosomal scale assembly of *Eimeria tenella* with reference-guided contig ordering and orienting in RaGOO and RagTag^3,28,29^.

### Genome structure prediction and annotations

**Fig. 2:**
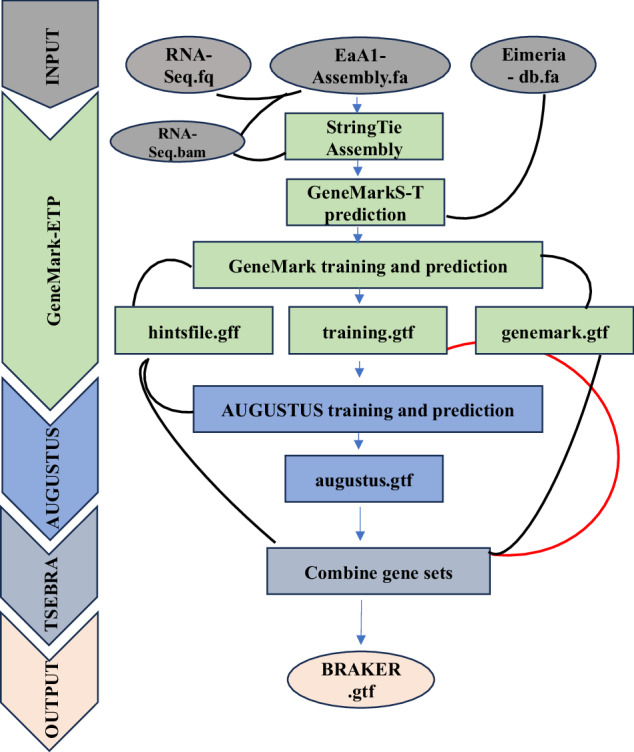
Diagrammatic representation of *E. acervulina* APU1 genome prediction workflow using the BRAKER3 pipeline with RNA-Seq and annotated proteins from related *Eimeria* species. The BRAKER3 pipeline uses GeneMark-ETP to integrate RNA-Seq and protein information to identify genes. The Genemark results are then used by AUGUSTUS to generate gene models and predict genes ab initio; AUGUSTUS accuracy improves with TSEBRA using RNA-Seq data as described in workflow. The results of each step are combined to produce the final BRAKER gene annotations.

Gene prediction and annotation seeks to infer function from coding and non-coding regions of the genome. We used the BRAKER3 pipeline (Fig. 2) that uses GeneMark-ETP to integrate RNA-Seq and protein information to identify genes. The GeneMark predictions were fed into AUGUSTUS for gene model development and prediction; AUGUSTUS accuracy was improved with TSEBRA using RNA-Seq and orthologous genes^30^ as described in the workflow (Fig. 2). We analysed our chromosomal scale genome assembly with RNA-Seq data from *E. acervulina*^31^ and employing protein data from related species^5^. The software was trained to predict genes across the genome assembly with data from a combination of bulk transcriptomic libraries (approximately 50 million reads from *E. acervulina* APU1 sporulated oocysts at 24 hours) as described (Tables 1, 6)^31^ and *Eimeria* species that are known to infect chickens^30^.

## Data Records

The NCBI Bioproject for this chromosomal scale assembly can be found at PRJNA913161 at accession number JAVIVJ030000000^32^. Raw data for this genome assembly can be found in NCBI at SRA:SRP414521^33^ with SRR31596726, SRR31596727 and SRR31596728 as the individual run numbers. The transcriptome used in the study was from publicly available NCBI SRA accession number SRP324149^34^ with samples GSM5385954, GSM5385955 and GSM5385956.

## Technical Validation

New sequencing technologies and chemistries enable more complete descriptions of complex genomes. Our *E. acervulina* genome assembly consists of 1.183 million high-quality long-reads (mean read length of 5,249 bases; mean quality score of 11.8) and 78 million high-quality NextSeq reads which were assembled by MaSuRCA^11^. This assembly produced an initial draft assembly of 51.70 Mb consisting of 114 contigs, N50 = 1,141,052 bases. We further constrained the assembly of these contigs employing Dovetail OmniC library reads of 17.5 Mb (~30 X coverage). Hi-C sequences were analysed by HiRise, to scaffold genome assemblies^12^ (Tables 1, 2). The improved Hi-C assembly provided 50 additional joins, and 2 breaks, to produced 50 scaffolds. These were re-scaffolded to fill gaps using evidence from ONT long reads, using recon and ntlink, to achieve chromosomal scale assembly. The longest scaffold thereby achieved was 6,840,217 bases, N = 50; 4,326,244 bases, L50 = 5. The shortest chromosomal scaffold was 845,591 bases. In total, 51,707,149 bases comprise our chromosomal scale genome assembly (Tables 2, 3)^35,36^. Assembly of mitochondrial and apicoplast organellar genomes was achieved from Nanopore and Illumina data employing the MitoHiFi^37^ and spades assemblers^38^, orientated with RaGOO and RagTag^28,29^. The assembled mitochondria and apicoplast genomes were 6,306 and 34,175 bp respectively (Table 3). These organelles were predicted and annotated to confirm the organelle specific genes.

**Table 2:** Feature of Assembled *E. acervulina* APU1.

Attributes	Assembly features
Country of origin	USA
Sequencing Technology	Hybrid (ONT/NextSeq/Hi-C)
Host /organism	Chicken/Parasites
Genome size (Mb)	52
Chromosomes	15
Longest chr sequence	6,840,217
Shortest chr sequence	845,591
N50	4,326,244
L50	5
Genome GC content (%)	48.15
Number of putative coding sequences	7,621
BUSCO Completeness; complete %	99%
CEG completeness: complete %	80%

**Table 3:** Statistics of *E. acervulina* APU1 genome length at chromosomal level.

Chromosome	Size
Chr15	6,840,217
Chr14	5,836,873
Chr13	4,856,252
Chr12	4,586,734
Chr11	4,326,244
Chr10	4,059,355
Chr9	3,320,098
Chr8	3,179,687
Chr7	3,001,917
Chr6	2,688,218
Chr5	2,090,460
Chr4	1,751,870
Chr3	1,705,884
Chr2	1,535,592
Chr1	845,591
Plastid; Apoplast	34,175
Mitochondria	6,306
Unplaced	1,067,636
Total	51,733,109

We annotated the chromosomal scale genome assembly using multiple approaches, ultimately identifying 7,621 genes. Of these, 6,305 genes were represented exactly once in the genome; the remaining 1,316 appeared to have multiple forms showing close similarity with homologues from related species of *Eimeria*^18^ (Fig. 1C). The individual software combination of prediction was 4,365 by AUGUSTUS, 1,426 GMST and 514 GeneMark^39–41^ (Fig. 2). Pfam annotation was identified for 4,647 genes and the GO Gene Ontology database identified molecular, biological, and functional classes for 1,962. The Cluster of Orthologous Genes (COG) analysis revealed primary functional categories for 30.12% (1,899/6,305)^42^ (Fig. 1D).

### Syntenic analysis and transcription factors

**Fig. 3:**
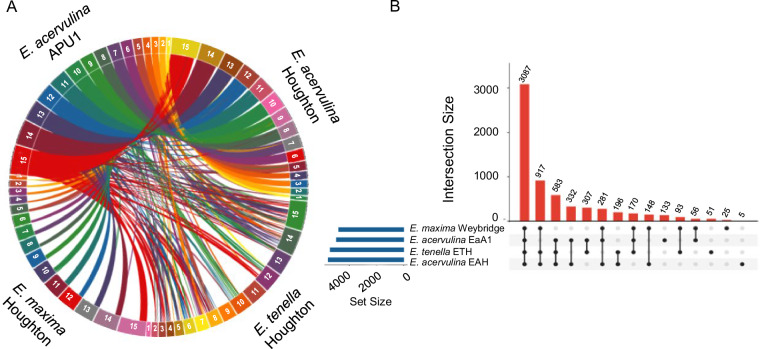
(**A**) Visualization of genome-wide synteny within (*E. acervulina* APU1 chromosomes vs *E. acervulina* Houghton) and between species (*E. acervulina* vs *E. maxima* vs. *E. tenella*) using Circa plot. *E. acervulina* APU1 and *E. acervulina* Houghton shared large genomic regions, whereas *E. maxima* and *E. tenella* were quite distant with only small, fragmented syntenic sections of their genomes. (**B**) UpSet analysis visualizes shared orthologs across groups of assemblies. Most orthologs were shared among all species examined. The *E. acervulina* assembly presented here had the highest number of unique orthologs of any assembly examined.

The syntenic relationship between *E. acervulina* APU1, *E. acervulina* Houghton, *E. maxima* Weybridge and *E. tenella* Houghton were analysed at the chromosomal level. We first constructed and orientated our chromosomal map to develop reference-guided contig ordering employing RaGOO^29^ and scaffolded these with RagTag^28^. We initially compared our assembly to those of the Houghton strain of *E. tenella*^26^, and with contig scale assemblies for the Houghton strains of *E. maxima* and *E. acervulina*^18^. We visualized contig synteny with Circa (http://omgenomics.com/circa) (Fig. 3A). As expected, the current assembly most closely matches that previously derived for the Houghton strain of *E. acervulina*^18^. We then examined evidence for collinearity among identified orthologous genes common to *E. acervulina, E. maxima*, and *E. tenella*^43^ (Fig. 3B). The comparative synteny between *E. acervulina* APU1 and *E. acervulina* Houghton was 44 Mb, whereas other *Eimeria* species were fragmented in small contig matches totalling 20 Mb with *E. maxima* and 32 Mb with *E. tenella*. We anticipate that fragmentation of syntenic regions could be driven by regions of high heterozygosity^3,18^.

We identified transcription factors that may control growth and developmental pathways. Transcription factors (TF) are key regulators of gene expression that bridge cell signalling and gene regulation^44^. TF such as the MYB (essential for cellular growth) and AP2 (ApiAP2, which plays a crucial role in regulating various stages of parasite life cycle transitions by controlling gene expression at different developmental stages) are involved in the regulation of key processes during parasite development and stage transformation^45^. Transcription factors orchestrate many signalling pathways in eukaryotes, and aberrant transcription factors underly numerous human diseases^46^. Transcription factors also regulate parasite gene expression^47^. We analysed the *E. acervulina* chromosomes to identify TFs based on similarities to documented TFs in the Animal TFDB database^48^. The predicted proteins were analysed with BLASTp, searching the TF database which consists of 270k TFs from 182 animal genomes and are classified into 73 families. We found a total of 43 TF when querying the entire dataset (Table 4), and used those defined for *Caenorhabditis elegans* (C. elegans) to annotate *E. acervulina* TF candidates. Doing so identified 21 TF, which agrees with 590 members that list these candidates and their annotation using Pfam terms (Table 5).

**Table 4:** The 43 Transcription Factor analysis using *E. acervulina* APU1 predicted proteins with database consists of large database of 270k TFs from 182 animal genome prediction and classified into 73 families.

SL. No.	EaA1 Genes	Family	full Sequence e-value	Best domain e-value	Domain Number
1	g90.t1	Germ_Cell_Nuclear_Factor-like	0.000071	0.000071	6
2	g206.t1	SNF2-rel_dom	1.00E-70	1.50E-70	1
3	g249.t1	TRAM_LAG1_CLN8	4.30E-40	6.90E-40	1
4	g370.t1	zf-C2H2	8.30E-30	0.00003	9
5	g398.t1	BTB	6.40E-16	3.90E-15	2
6	g432.t1	SNF2-rel_dom	7.10E-75	7.10E-75	3
7	g593.t1	CSD	1.80E-23	2.70E-23	1
8	g650.t1	GCFC	2.20E-32	2.20E-32	3
9	g655.t1	MOZ_SAS	7.00E-72	9.90E-72	1
10	g755.t1	Myb_DNA-binding	1.20E-11	2.30E-11	1
11	g1048.t1	Tub	5.90E-22	2.40E-15	5
12	g1136.t1	Myb_DNA-binding	0.0000037	0.0000077	1
13	g1384.t1	NFYB	4.80E-07	8.00E-07	1
14	g1418.t1	Germ_Cell_Nuclear_Factor-like	0.00002	0.000029	1
15	g1470.t1	Germ_Cell_Nuclear_Factor-like	0.000047	0.006	2
16	g1474.t1	SNF2-rel_dom	1.20E-64	1.20E-64	2
17	g2069.t1	zf-MIZ	4.80E-17	4.80E-17	2
18	g2139.t1	zf-LITAF-like	8.10E-15	8.10E-15	2
19	g2361.t1	HMG_box	0.000082	0.000082	2
20	g2464.t1	HMG_box	6.70E-08	0.00014	2
21	g2489.t1	Myb_DNA-binding	2.10E-17	1.20E-08	2
22	g2525.t1	Myb_DNA-binding	3.90E-11	1.00E-10	1
23	g2649.t1	zf-C2H2	3.10E-08	0.0018	2
24	g2831.t1	zf-C2H2	0.0000039	0.0000075	1
25	g3029.t1	Myb_DNA-binding	2.30E-18	6.80E-12	2
26	g3100.t1	BTB	7.70E-14	1.50E-13	1
27	g3181.t1	HMG_box	6.80E-22	1.00E-21	1
28	g3295.t1	Myb_DNA-binding	5.10E-10	1.10E-09	1
29	g3794.t1	HMG_box	5.60E-15	1.80E-14	1
30	g3800.t1	SNF2-rel_dom	4.00E-64	1.70E-63	1
31	g3941.t1	HMG_box	3.20E-08	1.00E-07	1
32	g3980.t1	Germ_Cell_Nuclear_Factor-like	0.000099	0.00012	1
33	g4157.t1	Myb_DNA-binding	1.60E-08	0.0031	3
34	g4235.t1	TRAM_LAG1_CLN8	4.60E-23	6.80E-23	1
35	g4348.t1	SNF2-rel_dom	4.60E-61	4.60E-61	3
36	g4499.t1	MOZ_SAS	1.20E-75	1.80E-75	1
37	g4832.t1	Germ_Cell_Nuclear_Factor-like	0.0000043	0.0000043	2
38	g4910.t1	Myb_DNA-binding	0.00017	0.00035	1
39	g5142.t1	HMG_box	1.10E-19	1.60E-19	1
40	g5175.t1	BTB	9.30E-07	0.0000025	1
41	g5200.t1	SNF2-rel_dom	1.40E-71	1.40E-71	2
42	g5211.t1	BTB	1.50E-14	3.80E-14	1
43	g5747.t1	zf-MIZ	2.60E-07	0.000036	3

**Table 5:** *E. acervulina* APU1 Transcription factor annotations using *Caenorhabditis elegans* (*C. elegans*) genome (closest worm) available in the database to annotate *E. acervulina* TF candidates.

SL. No.	EaA1 genes	C. elegans TF-genes	TF	Pfam annotation
1	g15.t1	ZK1067.2b.1	ZK1067.2	NA
2	g370.t1	Y55F3AM.14.1	zf-C2H2	zf-C2H2 Zinc finger, C2H2 type
3	g593.t1	F02E9.2a.1	lin-28 CSD	CSD ‘Cold-shock’ DNA-binding domain
4	g668.t1	F40G9.17.1	F40G9.17	ACBP Acyl CoA binding protein
5	g755.t1	Y113G7B.23 c.1	swsn-1 MYB	SWIRM SWIRM domain
6	g1152.t1	F49E10.5a.1	ctbp-1 THAP	2-Hacid_dh_C D-isomer specific 2-hydroxyacid dehydrogenase, NAD binding domain
7	g1603.t1	Y37E3.9.1	phb-1 Others	Band_7 SPFH domain/Band 7 family
8	g2069.t1	W10D5.3 c.1	gei-17 zf-MIZ	zf-MIZ MIZ/SP-RING zinc finger
9	g2201.t1	C32F10.5.1	hmg-3 HMG	Rtt106 Histone chaperone Rttp106-like
10	g2226.t1	F25H2.5.1	ndk-1 Others	NDK Nucleoside diphosphate kinase
11	g2489.t1	F38A5.13.1	dnj-11 MYB	DnaJ DnaJ domain
12	g3029.t1	D1081.8.1	cdc-5L MYB	Myb_Cef pre-mRNA splicing factor component
13	g3181.t1	Y48B6A.14.1	hmg-1.1 HMG	HMG_box HMG (high mobility group) box
14	g3794.t1	F40E10.2.1	sox-3 HMG	HMG_box HMG (high mobility group) box
15	g3984.t1	ZK783.4.1	baz-2 MBD	PHD PHD-finger
16	g4348.t1	Y116A8C.13a.1	SNF2	SNF2-rel_dom SNF2-related domain
17	g4447.t1	R05D3.11.1	met-2 MBD	SET SET domain
18	g4556.t1	C16A3.4.1	zf-C2H2	zf-C2H2_2 C2H2 type zinc-finger (2 copies)
19	g4575.t1	Y71G12A.3a.1	tub-2 Tub	ANAPC4_WD40 Anaphase-promoting complex subunit 4 WD40 domain
20	g5142.t1	F47D12.4a.1	HMG	HMG_box HMG (high mobility group) box
21	g5927.t1	F54F2.9.1	MYB	DnaJ DnaJ domain

### Stage-specific novel gene prediction and annotation

*Eimeria* oocysts undergo sporulation (maturation to the infectious stage by developing sporozoites in structures called sporocysts) under the influence of temperature, oxygen, and moisture^6,49^. The stage-specific transcriptomic profile shed light on the drivers and consequences of developmental change^50,51^. Chromosomal scale assembly supported by transcriptome profiles aid in genetic mapping and identifying transcriptional and post-transcriptional variations and Quantitative Traits Loci (QTL)^52^. We therefore supported gene annotation using transcriptome libraries produced from three biological replicates of oocysts of our sequenced strain of *E. acervulina* (APU1) as they underwent maturation for 24 hours^31^. Each biological replicate had over 50 million paired end reads (Table 6).

**Table 6:** *E. acervulina APU1* sporulation, stage specific (T24) novel gene prediction and annotation data (RNA-Seq) biological replicates statistics used in the analysis.

RNA-Seq	Left reads	Right reads	Overall read mapping rate
T24_rep1	57,344,162	57,344,162	94.30%
T24_rep2	34,319,244	34,319,244	94.50%
T24_rep3	54,059,755	54,059,755	93.20%
Avg.	48,574387	48,574,387	94.00%

**Fig. 4:**
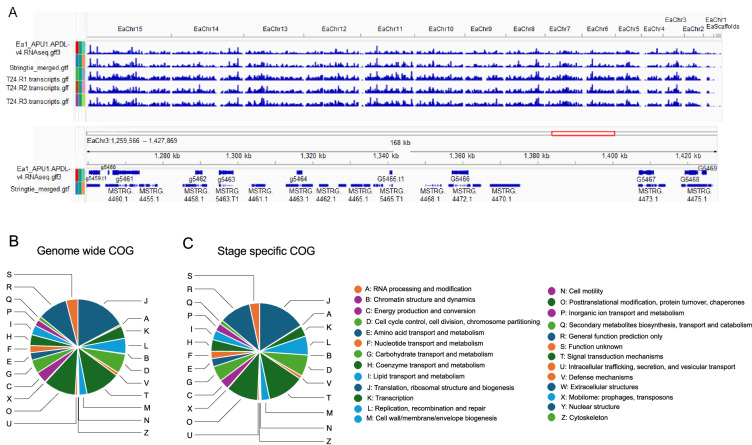
(**A**) Comparison of gene density across the genome for BRAKER3 gene predictions in this assembly and transcripts from RNAseq assembly replicates of *E. acervulina* sporulation at 24 hours.(Top view). The bottom view highlights differences between sporulation stage-specific (novel) transcripts and gene prediction for the chromosomal genome assembly. (**B**) Chromosomal scale gene predictions using the BRAKER3 pipeline uncovered 7,621 genes. Cluster of Orthologous Gene (COG) analysis revealed that 30.12% (1,899/6,305) single-copy genes have functional categories. (**C**) Stage-specific transcriptome assembly (T24) produced 9,325 genic-level transcripts; 31.64% (2,951 /9,325) of sporulation-specific transcript sequences were classified into COG functional categories.

The total transcriptomes revealed 47,556 exons in 9,761 transcripts. There were multiple transcripts for 3,601 of these loci which averaged 2.9 transcripts per locus. We compared the BRAKER3 predicted 7,621 genes from the assembled genome to the stage specific transcriptome assembly from the Cufflinks pipeline^53^. 44% (4,293) of Cufflinks and merged with transcripts with Stringtie^54^ compared common with BRAKER3 predictions. The 56% (5,468) of transcripts without BRAKER3 pipeline support due to reference-guided prediction (Table S2). The additional transcripts predicted from read-support (ORFs in RNA-Seq assemblies) were called cufflinks predicted novel transcripts^31,55^. These assembly transcripts were analysed for ORFs to find coding regions within transcripts (TransDecoder) with the minimum length criteria of 50 amino acids^56^. We retrieved 9,325 transcripts followed by Pfam and COG analysis (Table 5). Out of these 7,054 genes, Pfam identified with 4,054 having BRAKER3 annotation-supported genes and an additional 3,000 transcripts derived from *de novo* assembly of the sporozoite transcriptome (Fig. 4A and Table S2). The COG analyses classified 31.64% (2,951/9,325) sequences into COG functional categories (Fig. 4B,C and Table 7). These transcriptomes consider stage-specific transcripts (novel) associated with sporulation at 24 hours and analysed gene annotation and functional categories. The genome-wide transcriptome profile is depicted in the top view (Fig. 4A). The bottom view identifies stage-specific (novel) transcripts not found in the gene prediction and chromosomal scale genome, as judged by a multi-criterion approach for gene prediction and annotation; it uncovered 7,621 genes (Fig. 4B). Cluster of Orthologs (COG) analysis reveals that 30.12% (1,899/6,305) primary genes have functional categories. The stage-specific transcriptome assembly (T24) retrieved 9,325 genic-level transcripts out of 31.64% (2,951/9,325) sequences classified into COG functional category (Fig. 4C). These novel transcripts were annotated to understand their function, enabling their use to characterize for protein expression and immune responses^57^. Further in-depth analysis of parasite transcription factors, especially those that regulate virulence, may identify novel drug targets. Manipulation of these TFs may elucidate gene expression at various stages and improve gene annotation. We mapped all predicted and sporulation assembled transcripts to the chromosomal coordinates to establish their locations (Table S2).

**Table 7:** *E. acervulina* APU1, *E. acervulina* Houghton and *E. acervulina* sporulated (T24) Transcriptomes assembly COG annotation variation.

LETTER	EaA1	EAH	Sporulated RNA (T24)	DESCRIPTION
A	10	9	6	RNA processing and modification
B	3	3	1	Chromatin structure and dynamics
C	73	66	98	Energy production and conversion
D	126	144	211	Cell cycle control, cell division, chromosome partitioning
E	48	60	80	Amino acid transport and metabolism
F	46	38	72	Nucleotide transport and metabolism
G	88	100	152	Carbohydrate transport and metabolism
H	70	86	114	Coenzyme transport and metabolism
I	62	63	94	Lipid transport and metabolism
J	327	322	473	Translation, ribosomal structure and biogenesis
K	81	87	105	Transcription
L	99	142	186	Replication, recombination and repair
M	51	53	84	Cell wall/membrane/envelope biogenesis
N	6	4	7	Cell motility
O	208	208	315	Posttranslational modification, protein turnover, chaperones
P	41	50	76	Inorganic ion transport and metabolism
Q	24	35	39	Secondary metabolites biosynthesis, transport and catabolism
R	192	199	311	General function prediction only
S	76	71	100	Function unknown
T	225	238	362	Signal transduction mechanisms
U	14	12	17	Intracellular trafficking, secretion, and vesicular transport
V	20	27	33	Defense mechanisms
W	0	0	0	Extracellular structures
X	4	2	5	Mobilome: prophages, transposons
Y	0	0	0	Nuclear structure
Z	5	4	10	Cytoskeleton

In summary, this high-quality chromosomal scale genome assembly, combined with transcriptome annotation, provide insights into the genome evolution of *E. acervulina*. Accomplishing this chromosome-level genome will enable future studies of chromosomal rearrangement, investigations of pervasive gain or loss of genomic content, hasten identification of Quantitative Trait Loci (QTLs), and facilitate functional and evolutionary analyses of genes. Chromosomal orientation will hasten fine mapping of genes and examination of variation among closely related species. These studies may contribute to drug discovery and knowledge of host-parasite interactions that can impact control of parasitic disease.

## Supplementary information

Table S1.

Table S2.

## Data Availability

The pipelines for data processing were carried out in compliance with the established protocols of the bioinformatics software. We utilised USDA ARS SCINet HPC modules on a local high-performance server. The downstream processing analysis was processed using single and batch commends to finalise final output results presented in this study.
